# Antibiotic use and abuse: A threat to mitochondria and chloroplasts with impact on research, health, and environment

**DOI:** 10.1002/bies.201500071

**Published:** 2015-09-08

**Authors:** Xu Wang, Dongryeol Ryu, Riekelt H. Houtkooper, Johan Auwerx

**Affiliations:** ^1^ Laboratory of Integrative and Systems PhysiologyÉcole Polytechnique Fédérale de LausanneLausanneSwitzerland; ^2^ Laboratory Genetic Metabolic DiseasesAcademic Medical CenterAmsterdamThe Netherlands

**Keywords:** antibiotics, chloroplasts, doxycycline, environmental pollution, mitochondria, mitochondrial unfolded protein response, tetracycline

## Abstract

Recently, several studies have demonstrated that tetracyclines, the antibiotics most intensively used in livestock and that are also widely applied in biomedical research, interrupt mitochondrial proteostasis and physiology in animals ranging from round worms, fruit flies, and mice to human cell lines. Importantly, plant chloroplasts, like their mitochondria, are also under certain conditions vulnerable to these and other antibiotics that are leached into our environment. Together these endosymbiotic organelles are not only essential for cellular and organismal homeostasis stricto sensu, but also have an important role to play in the sustainability of our ecosystem as they maintain the delicate balance between autotrophs and heterotrophs, which fix and utilize energy, respectively. Therefore, stricter policies on antibiotic usage are absolutely required as their use in research confounds experimental outcomes, and their uncontrolled applications in medicine and agriculture pose a significant threat to a balanced ecosystem and the well‐being of these endosymbionts that are essential to sustain health.

Also watch the Video Abstract.

**Abbreviations**
cpDNA: chloroplast DNA
ETC: electron transport chain
mtDNA: mitochondrial DNA
nDNA: nuclear DNA
ROS: reactive oxygen species
UPRmt: mitochondrial unfolded protein response

## Introduction

**Figure 1:**
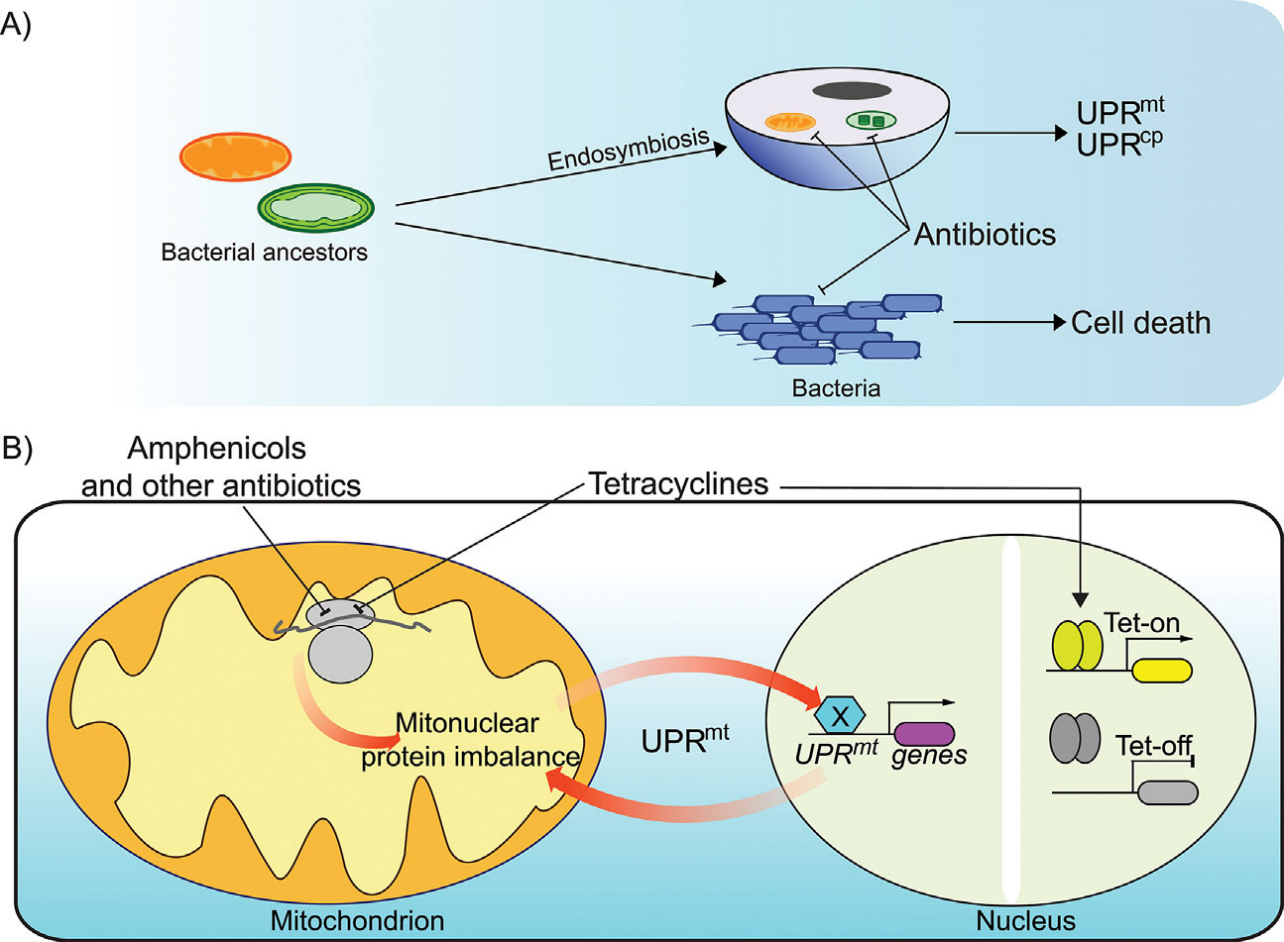
Conceptual figure illustrating the general idea showing the targeted effect of antibiotics. **A:** Antibiotics induce bacterial cell death meanwhile also interrupting the function of endosymbiotic organelles, mitochondria, and chloroplast, and generating a signal turning on the unfolded protein response pathways in eukaryotic cells. **B:** A schematic figure showing the targeted effect of tetracyclines on the Tet‐On/Tet‐Off system in nucleus and their adverse collateral effects on mitochondrial translation. Other antibiotics, such as the amphenicols, also have similar effects on the mitochondria. Using antibiotics that impair mitochondrial translation can induce a mitonuclear protein imbalance and lead to the mitochondrial unfolded protein response (UPR^mt^) pathway.

Mitochondria and chloroplasts are unique and subcellular organelles that have evolved from endosymbiotic α‐proteobacteria and cyanobacteria‐like prokaryotes, respectively (Fig. 1A) 1, 2. This endosymbiotic origin also makes these organelles vulnerable to antibiotics. Mitochondria and chloroplasts retained multiple copies of their own circular DNA (mtDNA and cpDNA), a vestige of the bacterial DNA, which encodes for only a few polypeptides, tRNAs and rRNAs 1, 3, 4. Furthermore, both mitochondria and chloroplasts have bacterial‐type ribosomes that are distinct from the 80S ribosomes in the cytoplasm; for instance, all chloroplasts contain 70S ribosomes, whereas animal mitochondria have 55–60S ribosomes and plant mitochondria have 70–80S ribosomes, depending on the species 5, 6.

Mitochondria are biochemical hubs contributing to a diversity of cellular events such as energy homeostasis, calcium homeostasis, thermogenesis, steroidogenesis, detoxification, inflammation, oxidative stress, cell death, and so on 7, 8, 9, 10, 11, 12. One of the major and well‐characterized roles of mitochondria is oxidative phosphorylation, the harvesting of energy contained in nutrients into adenosine triphosphate (ATP), the major energy currency molecule of life. The majority of the mitochondrial proteins are encoded by nuclear DNA (nDNA) and transcribed by the general transcriptional machinery into mRNAs that are transported into cytosol where they are translated into proteins by the cytoplasmic ribosomes. These nuclear encoded and mitochondria‐targeted proteins are imported in a co‐translation manner, folded, and assembled together with mtDNA‐encoded polypeptides that are translated by the specific translation machinery that resides in mitochondria 13, 14, 15. Assembly of complexes and supercomplexes of the mitochondrial electron transport chain (ETC) hence requires a stoichiometric match between nDNA‐encoded and mtDNA‐encoded polypeptides 16. An imbalance in the ratio between nuclear‐ and mitochondrial‐encoded proteins, termed the mitonuclear protein imbalance, inflicts a proteotoxic and metabolic stress on the mitochondria 17. The exposure of mitochondria to many stressors of a proteotoxic, energetic, osmotic, and oxidative nature explains the existence of multiple quality control mechanisms and adaptive pathways to maintain proper mitochondrial function, such as mitochondrial biogenesis, mitochondrial dynamics (fission/fusion), mitophagy, and the mitochondrial unfolded protein response (UPR^mt^) 18, 19, 20, 21, 22. Chronic overload of these quality control pathways is a major cause for mitochondrial dysfunction and contributes to the pathogenesis of mitochondrial diseases, ranging from rare inherited mitochondrial diseases to a palette of common age‐related disorders that include metabolic diseases, neurodegeneration, and cancer 8, 9, 23.

Chloroplasts are located in the cytoplasm of plant leaf cells as well as in micro‐ and macroalgae. There are three key subcompartments in the chloroplasts, including the chloroplast envelope, a double membrane, the stroma, and the thylakoid membranes, which form thylakoid vesicles 24. Chloroplasts fulfill many essential functions in plant cells, such as carbon assimilation through photosynthesis, nitrogen and sulfur metabolism, and biosynthesis of amino acids, chlorophyll, and fatty acids 24. Photosynthesis consist of light reactions and dark reactions 25. In light reaction, the energy of light is transformed into chemical energy in ATP and reduced nicotinamide adenine dinucleotide phosphate (NADPH) through the ETC and photophosphorylation on the thylakoid membrane. Then in the dark reaction also known as the Calvin cycle, the energy is transferred into sugars following carbon fixation in the stroma. Photosynthesis is sensitive to environmental perturbations, which may inhibit plant growth and influence crop yield, such as nutrient deficiency 26, 27, 28, 29, high light intensity 30, and diverse biotic and abiotic stresses 31, 32. In addition, impaired chloroplast translation can also significantly decrease chlorophyll content and efficiency of photosynthesis 5.

Antibiotics are antimicrobial organic substances that are produced from natural microorganisms or through industrial synthesis 33. Since the introduction of antibiotics for the treatment and prevention of bacterial infection about 7 decades ago, a wide variety of antibiotics have been used in human medicine as well as in agriculture for preventing or treating animal and plant bacterial infections 34. In addition, antibiotics are also used as feed additives for animals (mammals, birds, and fishes) to promote their growth 33. During the production and various application of such massive amount of antibiotics, they are released and can affect the environment. Whereas the public and scientific community has been mostly focusing on the influence of overuse or misuse of antibiotics on human health, there have been relatively few studies on the impact of antibiotics on ecosystems, especially on the kingdom Plantae. Here, we summarize recent achievements showing adverse effects of antibiotics on mitochondria and chloroplast function, which are off‐site targets of several antibiotics. The ultimate goal of this review is to inform the reader about why judicious antibiotic usage is required to protect our health and the ecosystem.

## Elevated antibiotic production increases potential of environmental release

**Figure 2:**
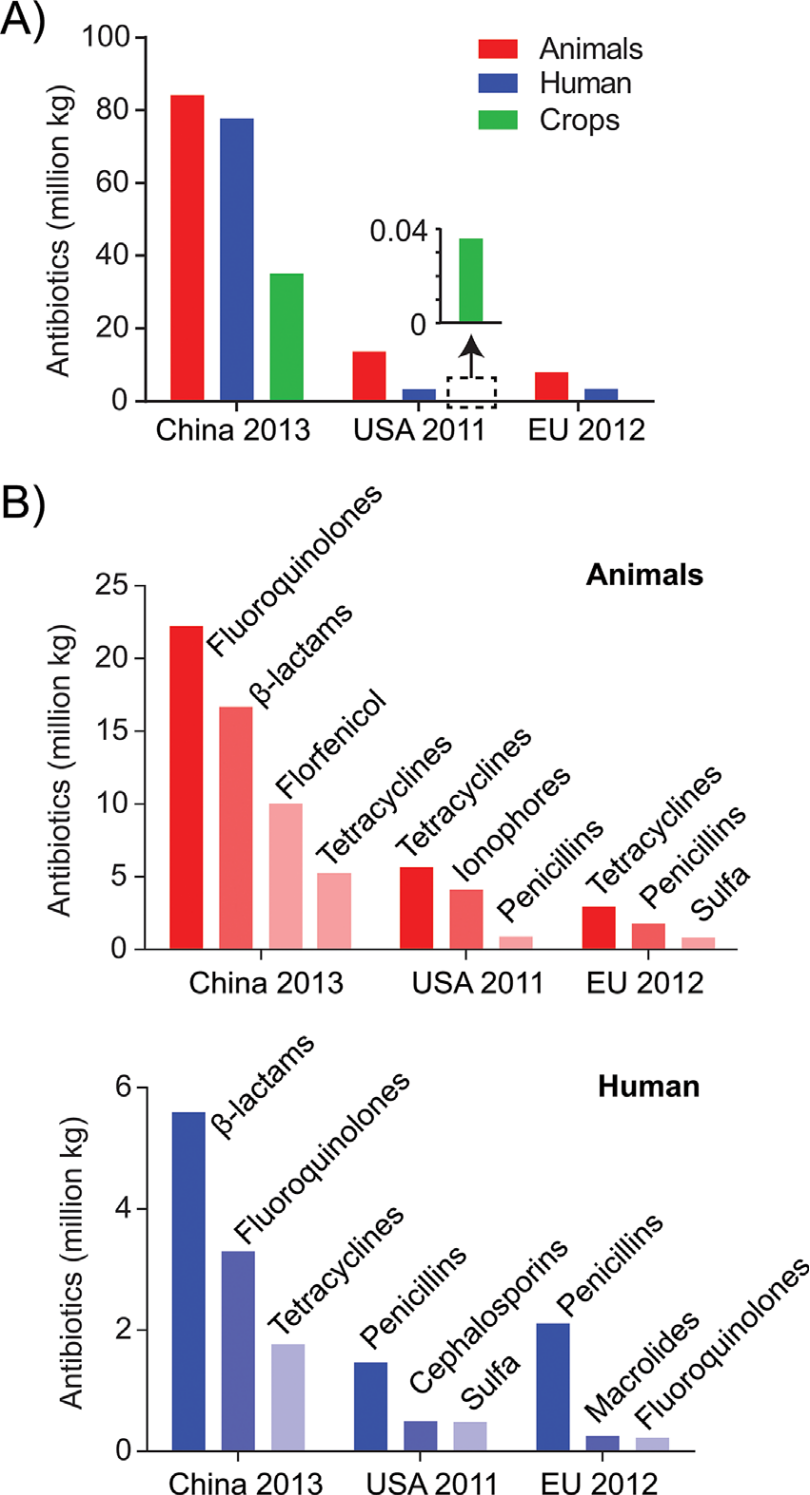
Usages of antibiotics in China, USA, and EU. **A:** Uses of antibiotics in human medicine, animals, and crop production. Data are from sales figures, which may not exactly reflect consumption. Usage data in crops in China were estimated from validamycin use, which consists of the majority of antibiotics consumption in China. A zoomed‐in chart shows antibiotics usage for crop production in the USA, which can hardly be seen in the main graph. Application of antibiotics in crop production is banned in the EU. **B:** Antibiotics most frequently used in humans and animals. The data on the antibiotics used in China are selected from those antibiotics that are frequently detected in the environment. Some antibiotics with relatively high use are not included due to their low detection frequencies 38. Although not in the top, a substantial amount of tetracyclines were sold in China, as indicated.

Due to the successful application of antibiotics in human medicine and especially in agriculture, antibiotic production has increased massively recently. From 2000 to 2010, human consumption of antibiotics increased by 36%, primarily in developing countries 35. According to Food and Drug Administration (FDA) reports 36, in 2011, ∼3.3 million kg of antibiotics were sold for human use and ∼13.6 million kg were sold for animal use in the USA, indicating that ∼80% of antibiotics were destined for agriculture applications (Fig. 2A). By 2013, the amount of antibiotics used in food‐producing animals increased to ∼14.8 million kg, increasing by 17% compared to the data in 2009 37. More critically, China produces and consumes the most antibiotics of all countries with an estimated ∼162 million kg of antibiotics being sold in China in 2013, of which almost half was used in animal feed 38 (Fig. 2A). It has been accepted that overuse or misuse of antibiotics may promote the development of antibiotic‐resistant bacteria 33. To solve this problem, the European Union (EU) has already forbidden the use of antibiotics to promote animal growth from 2006, and is trying to make antibiotics only available on medical or veterinary prescription 39. However, in 2012 there were still ∼8 million kg of antibiotics delivered to animals in EU countries 40 (Fig. 2A).

The particular antibiotics used vary in different countries. For instance in China, fluoroquinolones and β‐lactams are among the most commonly used antibiotics, while tetracyclines are also widely used for both humans and animals (Fig. 2B) 38. In the USA and EU, tetracyclines are the most commonly used antibiotics in animals, accounting for ∼40% of total antibiotics use (Fig. 2B) 36, 40.

Following the rising demand for animal protein, stockbreeding and aquaculture are developing rapidly, therefore, an unprecedented increase in the amount of veterinary antibiotics used is foreseen. According to an assessment of antibiotic consumption in livestock around the world using Bayesian statistical models, between 2010 and 2030, the global consumption of antimicrobials will increase by 67% in 2030 41. For countries such as Brazil, Russia, India, China, and South Africa (BRICS), the increase will be 99%, indicating that the double amount of antibiotics may be consumed in 2030 41. If such a prediction comes true, it will create even more challenges to control the release of antibiotics into the environment.

## Tetracyclines, lost in translation

Tetracyclines are broad‐spectrum polyketide antibiotics discovered from the *Streptomyces* genus of actinobacteria and they are acting by inhibiting bacterial protein synthesis (for detail see below). In the late 1940s, chlortetracycline and oxytetracycline were identified, and soon after tetracycline and doxycycline were synthesized 42, 43, 44. Before the wide awareness of antibiotic resistance in medicine, tetracyclines were the most common class of antibiotics used worldwide to treat infectious diseases and they are now still an available choice to manage certain diseases, such as acne, chlamydia infections, and Lyme disease 42. While their use in medicine has reduced, tetracyclines are still the most commonly used antibiotic class in veterinary medicine (sales: 2,943 tons in EU, 2012 40 and 6,515 tons in US, 2013 37). This wide use is explained because tetracyclines are relatively cheap and can be applied in the diet of farm animals at therapeutic levels to treat disease or at a subtherapeutic dose to improve animal growth rates 45.

In addition to their medical and veterinary applications, tetracyclines are also used as tool compounds in biomedical research to control the transcriptional regulator (Tet‐On/Tet‐Off system), to inhibit matrix metalloproteases and to label bone remodeling 46, 47, 48, 49, 50. Among those applications, the Tet‐On/Tet‐Off system is accounting for most of the research use of the tetracyclines. In Tet‐Off systems, the transactivator (tTA) protein, which is a fusion protein of the tetracycline repressor (TetR) of *E. coli* and the trans‐activating domain of VP16 of Herpes Simplex Virus, can be used to express genes placed under the control of a tetracycline‐response element (TRE). When tetracycline or a tetracycline derivative such as doxycycline binds to tTA protein, the tTA protein is released from the TRE and shuts down transcription. The Tet‐On system is basically operating in the opposite fashion to the Tet‐Off system and upon tetracycline binding to the rTA protein it allows it to interact with the TRE and for transcriptional activation to occur 51. Although the Tet‐On/Tet‐Off system is exquisitely flexible to study gene function without apparent developmental defects in vivo and in vitro, several studies have warned about the potential detrimental and confounding effects of the use of tetracyclines (Fig. 1B).

Tetracyclines occupy the A‐site of the bacterial 30S ribosomal subunit and inhibit bacterial polypeptide synthesis by sterically blocking the recruitment of the aminoacyl‐tRNA to the bacterial ribosome 43, 44, 52. Ribosomes are biological machines composed of RNAs and proteins that are responsible for protein synthesis and that are conserved across kingdoms. Many antibiotics that are clinically approved and widely used in research, such as the tetracyclines, also have powerful inhibitory effects on mitochondrial ribosomes and protein synthesis, which is not surprising given the proteobacterial origin of mitochondria 53. Tetracyclines are potent inhibitors of mitochondrial translation in rat heart and liver (IC_50_ = 2.1 µM) 54. Along with this, several studies reported that tetracyclines reduce cell proliferation in various human cell lines and cause many adverse effects in thymocytes and HepG2 cells 55, 56. Very recently, we have demonstrated that doxycycline disturbed mitochondrial proteostasis through the induction of an imbalance between mitochondrial and nuclear protein production, aka the mitonuclear protein imbalance 17. This effect was observed in human embryonic kidney (HEK) 293 and HeLa cells as well as in mouse hepatoma Hepa1‐6 and hypothalamic GT1‐7 cell lines, and was present even at low concentrations (∼0.5 µg/mL) 17, 57 (Fig. 1). In mouse and human cells, the induction of mitonuclear protein imbalance was accompanied by major changes in mitochondrial function (e.g. oxygen consumption rate), mitochondrial dynamics (e.g. induced fragmented mitochondria), as well as marked repression of ∼10% of nuclear genes 57. Moreover, doxycycline impaired development and mitochondrial function in the nematode *C. elegans* and the fruit fly *D. melanogaster* 57. Similarly, C57BL/6J mice that drank water containing doxycycline (50 or 500 mg/kg/day) for 14 days displayed a similar mitonuclear imbalance and mitochondrial dysfunction. Furthermore, energy expenditure was reduced in doxycycline‐treated mice, compared to the controls receiving amoxicillin, which does not prevent bacterial/mitochondrial translation but rather targets bacterial wall synthesis. Also in plants, 25 mg/L doxycycline severely repressed growth of *Arabidopsis* seedlings, significantly decreased oxygen consumption, and reduced mitochondrial translation, indicative of repressed mitochondrial function. In summary, doxycycline altered mitochondrial function in immortalized mammalian cell lines, worms, fruit flies, mice, and across kingdoms in plants 57.

## Other antibiotics that impact on mitochondria

Given the body of evidence for the endosymbiotic theory 53, and the similarity of ribosomal machinery between bacteria and mitochondria, it is not surprising that, besides tetracyclines, also other antibiotics that target bacterial protein synthesis can affect mitochondrial protein synthesis (Fig. 1, and Table 1). Antibiotics of the families of the aminoglycosides, amphenicols, lincosamides, macrolides, oxazolidinones, streptogramins – all known as inhibitors of bacterial protein synthesis – also block mitochondrial polypeptide synthesis, often without a parallel effect on the cytoplasmic ribosome. Conversely, the antibiotic cycloheximide, which is an antifungal agent, does not inhibit bacterial and mitochondrial protein synthesis but prevents eukaryotic cytoplasmic polypeptide synthesis 58.

**Table 1:** Antibiotics affecting bacterial protein synthesis and human health

Class	Name	Target	Reported side effects	References
Aminoglycosides	Amikacin, Dibekacin, Gentamicin, Kanamycin, Neomycins, Streptomycin, Tobramycin	Peptide elongation at the bacterial 30S ribosomal subunit	Kidney injury, ototoxicity, and vestibular toxicity	60
Amphenicols	Chloramphenicol, Thiamphenicol	Protein elongation by overlapping with the binding site at the A‐site of 50S ribosomal subunit	Aplastic anemia, bone marrow suppression, neurotoxicity	62, 63
Macrolides	Azithromycin, Carbomycin A, Clarithromycin, Erythromycin	Peptide‐bond formation and ribosomal translocation	Myopathy, QT prolongation, nausea	65
Oxazolidinones	Eperezolid, Linezolid, Posizolid, Radezolid, Sutezolid	Peptide‐bond formation by blocking tRNA binding at the A‐site of 50S ribosome	Nausea, bone marrow suppression, lactic acidosis	54, 61
Streptogramins	Pristinamycin, Quinupristin/dalfopristin, Virginiamycin	Protein elongation at the A‐ and P‐sites of 50S ribosome	Nausea, myalgia, arthralgia	68
Tetracyclines	Doxycycline, Chlortetracycline, Lymecycline, Meclocycline, Minocycline, Tetracycline	Polypeptide synthesis by sterically blocking the recruitment of the aminoacyl‐tRNA at the A‐site of the bacterial 30S ribosomal subunit	Phototoxicity, secondary intracranial hypertension, teeth discoloration, steatosis, liver toxicity	53, 54, 55, 56

References are reporting an effect on mitochondria, except for 68 which refers to chloroplasts.

Aminoglycosides are a group of antibiotics that include amikacin, dibekacin, gentamicin, kanamycin, neomycins, streptomycin, and tobramycin. Aminoglycosides induce the misreading and premature termination of mRNA translation through perturbing peptide elongation at the bacterial 30S ribosomal subunit 59. Several of the side effects of the aminoglycosides, such as kidney injury, ototoxicity, and vestibular toxicity, are hallmarks of mitochondrial toxicity; especially, the ototoxicity has been associated with mitochondrial ribosomal dysfunction 60. Chloramphenicol, a member of the amphenicol class that was isolated from *Streptomyces venezuelae,* binds to the 23S rRNA of the 50S ribosomal subunit and prevents bacterial protein elongation by overlapping with the binding site at the A‐site 52, 61. Likewise, chloramphenicol and thiamphenicol shut down mitochondrial translation 62, 63. In mammalian cells treated with either chloramphenicol or thiamphenicol, mtDNA‐encoded proteins (e.g. MT‐ND1, MT‐CO1, and MT‐CO2) were dramatically reduced while nDNA‐encoded respiratory gene transcripts (e.g. *ATP5A1*, *COX5A*, and *COX8A*) 62 and proteins (e.g. ATP5A) were increased [unpublished results of the authors]. Finally, the amphenicol‐induced mitonuclear imbalance between nDNA‐ and mtDNA‐encoded cellular respiratory proteins induces the mitochondrial unfolded protein response, typified by the induction of HSP60, Mortalin, LONP1, and CLPP (see 17 and unpublished results of the authors) (Fig. 1). Also in *C. elegans,* chloramphenicol induced a mitonuclear imbalance, activated the UPR^mt^ and reduced mitochondrial respiration 17.

Erythromycin was the first of the macrolide antibiotics discovered in 1952. Macrolides including azithromycin, carbomycin A, clarithromycin, and erythromycin bind within the exit tunnel of the bacterial ribosome and perturb peptide‐bond formation and ribosomal translocation 52, 64, and consequently also have an inhibitory action on mitochondrial protein synthesis 65. Oxazolidinones are a class of antimicrobial agents that prevent peptide‐bond formation by blocking tRNA binding at the A‐site of the bacterial 50S ribosome 52. The adverse effects of oxazolidinones reflect their deleterious action on mitochondria, and include hyperlactemia, metabolic acidosis, and peripheral neuropathy 54, 66. Pristinamycin, quinupristin/dalfopristin, and virginiamycin are of the family of the streptogramins isolated from *Streptomyces pristinaespiralis*, and inhibit bacterial protein synthesis by occupying on the A‐ and P‐sites of 50S ribosome 52, 67. While the effect of streptogramins on mitochondrial translation and function are not yet clearly demonstrated, inhibitory actions of virginiamycin on protein synthesis in chloroplasts were reported 68.

In addition to antibiotics directly targeting the mitochondrial ribosome, several studies demonstrated that a number of antibiotics, including quinolones (i.e. ciprofloxacin), β‐lactams (i.e. ampicillin), and aminoglycosides (i.e. kanamycin), induce oxidative stress via the depletion of the primary reducing equivalent NADH in bacterial, as well as, in mammalian cells 69, 70, 71. The fact that highly deleterious hydroxyl radicals and the NAD^+^/NADH ratio was also increased after the addition of antibiotics to wild‐type *E. coli*, led to the hypothesis that an oxidative damage‐induced cell death pathway could underpin the bactericidal effects of the antibiotics 71. Interestingly, in 6 to 8‐week‐old wild‐type female FVB/NJ mice, a 16‐week treatment of ciprofloxacin (12.5 mg/kg/day), ampicillin (28.5 mg/kg/day), or kanamycin (15 mg/kg/day) also induced oxidative stress in blood and mammary gland 69. Evaluating the effect of antibiotics on NAD^+^ and NADH levels and mitochondrial function, hence, warrants future investigation.

This short literature review actually underscores that several antibiotic classes affect mitochondrial activity, which is not so surprising given the endosymbiotic nature of these organelles (Fig. 1B). Future studies should define the mechanisms how these antibiotics achieve these mitochondrial effects (mitochondrial translation, oxidative stress, NADH depletion, or other mechanisms), potentially leading to the development of new antibiotics that are safer antimicrobials and cleaner research tools and which leave organelle function intact.

## Antibiotics reach plants through multiple pathways

**Figure 3:**
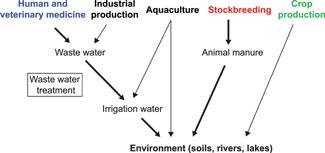
Sources and pathways of how antibiotics are released into the environment. Antibiotics reach the environment through multiple ways, the main pathways beginning from human and agricultural use are highlighted. The thickness of the arrows reflects the relative importance of the pathways.

There are mainly three ways for the environmental release of antibiotics: (i) after therapeutic use in human and veterinary medicine; (ii) after agricultural use, i.e. for growth promotion in stockbreeding and aquaculture, and therapeutic and preventive use in plant production; and (iii) non‐intentional release from industrial production 33, 72, 73 (Fig. 3). According to the statistical data from China, USA, and EU, most of the antibiotics are used in humans and farm animals (Fig. 2A). Previous reports showed that due to incomplete absorption, 30–90% of antibiotic doses given to humans and animals may be released in the urine and feces after medication 33. Therefore, urban wastewater, biosolids, and animal manure contribute most to the environmental release of antibiotics. Among different antibiotic classes, tetracyclines are easily dissolved in water and could persist in soil for over 1 year, making them the most frequently detected and major antibiotic released into the environment 33, 74. For example, soil residues of tetracyclines have been detected to be up to 307 mg/kg in China 75 and 198.7 mg/kg in Germany 76, while the natural background level of total antibiotics were normally less than 5 mg/kg 33.

Since the 1950s, antibiotics have been applied to control bacterial infection in agricultural plants, such as high‐value fruit, vegetable, and ornamental plants 34. In the USA, antibiotics applied to plants account for only 0.26% (∼36,000 kg) of total agricultural utilization in 2011, which are mainly confined to use in orchards 77 (Fig. 2A). In China, however, it is estimated that more than 80 million kg of antibiotics, fungicides, and insecticides are produced per year for crops 78, from which validamycin is the most commonly used antibiotics (∼35 million kg produced per year, Fig. 2A) 79, for the control of sheath blight of rice through inhibiting the trehalase activity in fungal pathogens 80. Although validamycin also inhibits trehalase in plants and leads to alterations in carbohydrate allocation, its potential side effects on plant growth in the environment has not been fully evaluated 81.

## Antibiotics induce phytotoxicity in the environment

Previous studies showed that terrestrial and aquatic plants could take many kinds of antibiotics up from the polluted environment 82, 83. In general, the uptake and effects on plants varies and depends on the antibiotic and plant species, as well as soil and water characteristics 82, 84. In most studies, antibiotics consistently showed toxic effects on farm plants, such as ryegrass 85, maize 86, alfalfa, carrot, lettuce 84, cucumber, and rice 87. Some plants are extremely sensitive to antibiotics; for example, root elongation in carrot seedlings was reduced by 50% at 0.2 mg/L of tetracycline (EC_50_) 84. In our recent study, 1 mg/L of doxycycline can significantly inhibit root hair growth in *Arabidopsis* 57. More cases of antibiotic toxicity on plants can be found in some recent reviews 33, 82.

In some plant species, low concentration of antibiotics may oppositely improve plant growth 75, 88, 89, 90 probably due to “hormesis,” an adaptive and mostly beneficial response to low levels of toxins. However, the beneficial range of antibiotics is usually quite narrow, for example, 0.5–5 mg/L of tetracycline can stimulate cell mitotic division and growth of wheat seedlings, while concentrations higher than 5 mg/L cause adverse effects on wheat growth 75.

## Plant chloroplasts and mitochondria are vulnerable to antibiotics

**Figure 4:**
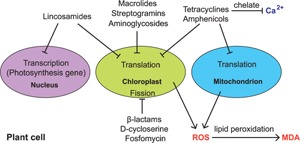
Schematic diagram of the impact of antibiotics on a hypothetical plant cell. For simplicity, all factors are presented in the same plant cell. Arrows indicate positive regulations and bars mean negative regulations. ROS, reactive oxygen species; MDA, malondialdehyde, is an end product of lipid peroxidation.

How can antibiotics inhibit plant growth? Many relevant studies focused on the impact of antibiotics on photosynthesis and oxidative stress response in plants 75, 86, 91, 92, 93, 94. The bacterial origins of chloroplasts and mitochondria explain why they may be vulnerable to antibiotics (Figs. 1 and 4). In the moss *Physcomitrella patens* and the green algal *Closterium*, studies showed that D‐cycloserine, fosfomycin, and β‐lactam antibiotics (e.g. ampicillin) interfered with peptidoglycan biosynthesis and inhibit chloroplast division, causing cell division inhibition and cell death 95, 96. However, chloroplasts in vascular plants have lost the peptidoglycan layer in their envelopes, making vascular plants insensitive to these antibiotics 96.

Chloroplast translation is the main target of many antibiotics because of its similarity to the prokaryotic translational machinery 97. The inhibitory effect of streptogramins (virginiamycin) on protein synthesis in chloroplasts are already mentioned above 68. Aminoglycoside antibiotics (e.g. streptomycin, kanamycin, neomycin, gentamicin) can enter chloroplast through an iron transporter (MAR1, multiple antibiotic resistance 1), located on chloroplast membrane; in the chloroplast these antibiotics inhibit chloroplast translation by targeting ribosomal 16S and 23S rRNA 98. Another study showed that aminoglycosides (e.g. spectinomycin), lincosamides (e.g. lincomycin), and macrolides (e.g. erythromycin) inhibited translation in the chloroplast without direct effects on cytoplasmic protein synthesis 93. Lincomycin can also repress the transcription rate of some nuclear encoded photosynthesis‐related genes, such as *Lhcb* (Chlorophyll a‐b binding protein) and *RbcS* (Ribulose bisphosphate carboxylase small chain) 93, perhaps due to a signal originating from dysfunctional chloroplasts. Tetracyclines and amphenicols (e.g. chloramphenicol) also repress photosynthesis 75, 86, 91, 99. Although some studies showed that tetracyclines at high concentrations (500 mM) can quickly inhibit chloroplast translation by binding 16S rRNA and blocking the entry of amino‐acylated tRNA into the A site of the 70S ribosome 97, in our hands chloroplast translation is ∼10‐fold less sensitive to inhibition than mitochondrial translation 57. Despite high similarity between chloroplast and prokaryotic translational machinery, the exact targets of some antibiotics in the chloroplast remain to be determined.

Besides these effects on chloroplasts, early work described that tetracyclines, as well as chloramphenicol, inhibit translation of proteins encoded by mtDNA, but not by nDNA 100. In our recent study 57, plants displayed marked mitonuclear protein imbalance, implying specific inhibition of mitochondrial translation. However, the direct target of tetracyclines in plant mitochondria remains unknown. Moreover, lipid peroxidation and reactive oxygen species (ROS) were frequently found in plants exposed to tetracyclines 75, 86, 94. Since mitochondria are one of the main ROS sources in plants 101, it will be interesting to evaluate in the future whether mitochondria contributes to ROS accumulation under antibiotic stress.

In addition, it was reported that chlortetracycline uptake leads to reductions in levels of intracellular calcium due to chelation (Fig. 4). In turn, reduced calcium changes overall patterns and levels of protein synthesis and induces toxic effects 102. Therefore, the mechanisms that antibiotics employ to repress plant growth seem to be multiple and not limited to the ones mentioned above. Further studies of the interactions of various antibiotic and plant species will hence be invaluable to fully understand the impact of environmental released antibiotics on plants.

## Conclusion and outlooks

Since 1911 when the first antibiotic, arsphenamine was discovered, antibiotics took the center stage of human and animal medicine and saved many lives. Nowadays antibiotics are not only used for medical/veterinary indications, but also in biomedical research as well as for agricultural applications. They are these “non‐medical,” often uncontrolled applications that pose particular threats.

As to the use for research purposes, antibiotics such as penicillin, streptomycin, and gentamicin are essential to prevent microbiological contamination in eukaryotic cell culture. Ampicillin, kanamycin, puromycin, hygromycin, and geneticin (G418) are the most popular antibiotics for selecting specific cells expressing or harboring transduced genes (typically the antibiotic‐resistance gene together with the gene‐of‐interest). In addition, the Tet‐On/Tet‐Off system using tetracyclines allows the delicate temporal and spatial control of gene expression avoiding confounding or secondary effects, caused by chronic overexpression or knockdown of a gene of interest. However, the use of antibiotics is a double‐edged sword as exemplified by the induction of mitochondrial proteotoxic stress by doxycycline, which alters not only mitochondrial dynamics and function but also global gene expression patterns in immortalized cell lines, worms, fruit flies, mice, and plants 56, 57. To avoid such undesired confounders caused by organellar – mitochondrial and chloroplast – mistranslation, researchers have to be well aware of the potential effects of antibiotics on mitochondrial and chloroplast function, gene expression, and cell proliferation. Future research should also define cleaner research tools that do not affect the function of organelles, such as mitochondria and chloroplast that are vital for all aspects of physiology.

Global antibiotics production and consumption are still increasing year by year, and pose a potential threat not only to human health but also to the delicate homeostasis of our ecosystem. Plants show differential susceptibility to antibiotics, and even in the same species genotypic differences may lead to significant divergence in susceptibility 103, 104, alerting us to take care of their health and to avoid non‐natural selection that may occur in heavily polluted regions. To protect plants, animals, and humans in our ecosystem, instead of restricting antibiotics use, we may select or design antibiotics that do not target components of organelles. Therefore, more efforts to study the relationship between antibiotics and endosymbiotic organelles, such as the mitochondrion and chloroplast, are warranted.

The authors report no conflicts of interest.
